# Bcr-Abl Tyrosine Kinase Inhibitors in the Treatment of Pediatric CML

**DOI:** 10.3390/ijms21124469

**Published:** 2020-06-23

**Authors:** Francesca Carofiglio, Antonio Lopalco, Angela Lopedota, Annalisa Cutrignelli, Orazio Nicolotti, Nunzio Denora, Angela Stefanachi, Francesco Leonetti

**Affiliations:** Dipartimento di Farmacia Scienze del Farmaco Università degli Studi di Bari “Aldo Moro”, via Orabona 4, Bari 70125, Italy; francescacarofiglio94@gmail.com (F.C.); antonio.lopalco@uniba.it (A.L.); angelaassunta.lopedota@uniba.it (A.L.); annalisa.cutrignelli@uniba.it (A.C.); orazio.nicolotti@uniba.it (O.N.); nunzio.denora@uniba.it (N.D.)

**Keywords:** chronic myeloid leukemia, tyrosine kinase inhibitors, pediatric age, imatinib, dasatinib, nilotinb, ponatinib, formulation

## Abstract

The therapeutic approach to Chronic Myeloid Leukemia (CML) has changed since the advent of the tyrosine kinase inhibitor (TKI) imatinib, which was then followed by the second generation TKIs dasatinib, nilotinib, and, finally, by ponatinib, a third-generation drug. At present, these therapeutic options represent the first-line treatment for adults. Based on clinical experience, imatinb, dasatinib, and nilotinib have been approved for children even though the studies that were concerned with efficacy and safety toward pediatric patients are still awaiting more specific and high-quality data. In this scenario, it is of utmost importance to prospectively validate data extrapolated from adult studies to set a standard therapeutic management for pediatric CML by employing appropriate formulations on the basis of pediatric clinical trials, which allow a careful monitoring of TKI-induced adverse effects especially in growing children exposed to long-term therapy.

## 1. Introduction

Protein kinases (PK) enable the phosphorylation of hydroxyl groups of tyrosine, serine, and threonine residues. Based on this signaling, a cascade of molecular events is activated to promote a number of biochemical actions responsible for cells proliferation, survival, and functioning. There are two main classes of tyrosine kinases. The first class is made up by receptors tyrosine kinases (RTK) linked to transmembrane receptors. The second class is known as cytoplasmic non-receptor tyrosine kinases (NRTK) [1]. Receptors activated by a vascular endothelial growth factor (VEGF) and a platelet-derived growth factor (PDGF) and overexpressed in sarcoma, Breakpoin cluster region-Abelson Kinase (BCR-ABL) in myeloid leukemia, and JAK (Janus Kinases) are of oncology relevance as far as RTK and NRTK are concerned [1].

The overexpression or mutation of tyrosine kinases (TK) is a hallmark of cell cycle dysregulation often anticipating the tumor onset. For these reasons, selective TK inhibition is considered a target therapy in cancer [2].

Chronic myeloid leukemia (CML) is a myeloproliferative disorder, characterized by an abnormal granulocyte cells proliferation determining a high increase of white blood cell count in addition to a spleen enlargement (splenomegaly). The CML pathogenesis stems from the Philadelphia chromosome, discovered by Peter Noweel in 1960. It arises from a translocation of the TK ABL (Abelson) gene from chromosome 9 to chromosome 22, on the BCR gene (breakpoint cluster region). The translocation generates the oncogenic BCR-ABL1 fusion gene in hematopoietic stem cells, which encodes an abnormal protein, with constitutive TK activity, responsible for proliferative and anti-apoptotic signals. The occurrence of BCR-ABL protein kinases was observed in more than 90% of CML patients [3].

The BCR-ABL targets are downstream pathways including RAS, PI3K/AKT, and JAK/STAT that address the transformation of healthy cells toward neoplastic cells responsible for CML pathogenesis.

CML has three clinical phases. The first is the chronic phase (CP) without subjective symptoms after 3–5 years from diagnosis but a high white blood cell and platelet count. The second is the accelerated phase (AP) with an incremented differentiation of abnormal granulocytes. The third phase consists in the blast crisis (BC) with an increase of undifferentiated blasts. Patients in the chronic phase can be treated with tyrosine kinases inhibitors (TKIs). Unfortunately, accelerated and blast phases are not responsive to TKIs likely because their progression is not affected by BCR-ABL.

The 3D structure of BCR-ABL kinase, like other tyrosine kinases, is characterized by two lobes, including the N-terminal and the C-terminal one, connected by a short peptide strand, known as a hinge region. ATP (adenosine triphosphate) binds to the fissure between the lobes through two hydrogen bonds with a residue of Glu316 and Met318.

Moreover, in the rear portion of the ATP binding pocket, there is a residue of Thr315 with a key role for selectivity. This residue is called “gatekeeper” because of a resistance phenomenon associated with its related single point mutations.

The Bcr-Abl activation is determined by a flexible protein segment in the N-terminal lobe, called the activation loop. In its active state, it adopts an open conformation with the amino acid triad Asp-Phe-Gly (DFG, corresponding to 381–383 residues in Abl) directed toward the ATP binding site. This is the DFG IN conformation, whichh allows ATP to approach the binding site, where the Phe residue of the triad is located.

**Figure 1:**
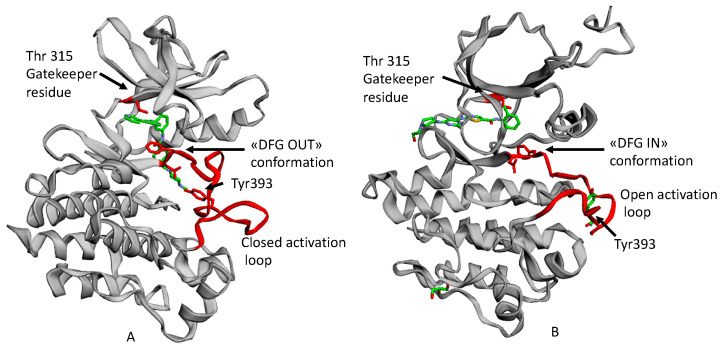
An example of ATP competitive inhibitors: (**A**) X-ray solved structure of Abl kinase domain in complex with Imatinib (PDB code: 1IEP) with DFG outconformation and closed activation loop. (**B**) X-ray solved structure of Abl Kinase domain with Dasatinib (PDB code: 2GQG) with DFG in conformation, open activation loop [5].

When the activation loop is closed as it is in the inactive state, the Asp-Phe-Gly amino acid triad adopts the DFG out conformation (Figure 1) [4].

## 2. CML Therapeutic Approach

In the past decades, the first therapeutic approach to CML involved the use of ordinary chemotherapeutical drugs (such as a busulfan, hydroxyurea, cyclophosphamide, and vincristine), which is followed by allogeneic hematopoietic stem cells transplantation (allo-HSCT). This is considered a potentially curative procedure for a variety of hematological malignancies in order to reconstitute hematopoiesis [6].

However, allo-HSCT is limited by the availability of donors, transplant-related mortality, and early morbidity, so that it is considered a third-line treatment for most pediatric cases. Therefore, Bcr-Abl inhibitors may be accounted as a targeted therapy toward CML, avoiding all the side effects associated with canonical chemotherapy, and graft-versus-host disease, which is one of the major complications of allogeneic hemapoietic cell transplantation (HCT).

Currently, Imatinib is not the only TK inhibitor approved for patients with CML-CP (CML-Chronic Phase) including pediatric patients, but it remains the first-line therapy for patients with CML-AP (CML-Accellerate Phase) and CML-BC (CML-Blast Crisis).

**Figure 2:**
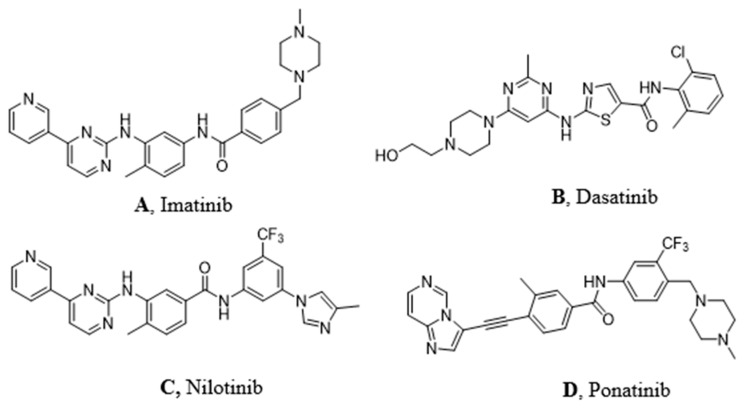
Molecular structures of the principal BCR-ABL TKIs used for treating pediatric chronic myeloid leukemia (CML): (**A**) Imatinib; (**B**) Dasatinib; (**C**) Nilotinib; (**D**) Ponatinib.

Imatinib (Figure 2A) was discovered in 1992 as a selective inhibitor of Bcr-Abl TK. It is able to link to the kinase in its inactive form preventing the ATP binding (Figure 1A). It was approved in 2001 by FDA as first-line therapy for CML patients by obtaining optimal results compared to those who had been treated with hydroxyurea and IFN-α in terms of CHR (Complete Hematological Response), CCR (Complete Cytogenetic Response), and MMR (Major Molecular Response). Unfortunately, the appearance of resistance phenomena required the development of a second generation of TKI [6].

### 2.1. Resistance and Intolerance to Imatinib

The urgent need to develop a second-generation of TK inhibitors was principally due to the resistance and the intolerance of some patients to imatinib. A patient can be defined resistant to imatinib when presenting an increasing white blood cell or platelets counts that prove a primary resistance or a hematologic relapse. Moreover, resistance to imatinib consists of suboptimal cytogenetic or molecular response or failure, progression to AP/BC, reappearance of Ph+ bone marrow cells following achievement of complete cytogenetic response (CCR) and, at least, increase of more than 30% in Ph+ cells in peripheral blood or bone marrow, or loss of a molecular response. Frequently, resistance is associated with emerging mutations in some BCR-ABL amino acid residues. We can count 12 residues with risk of mutations that can be classified in resistant (T315I), less sensitive (Y235H, F359V, E255K), and sensitive (all other mutations except those mentioned before) [7].

Other resistance phenomena such as P-gp (Glycoprotein P) efflux pump overexpression, OCT1 (Organic Cation transporter) reduced expression, and the activation of alternating oncogenic pathways (Src (proto oncogene Sarcome tyrosin kinase), PI3K (Phosphoinositide 3-kinases), PDGFR, KRAS (Kirsten rat sarcoma Kinase), and JAK2) must be reminded [8,9].

Furthermore, intolerance is related to imatinib side effects due to its off-target activity. Among all, we can remember the increase of blood bilirubin, headache, rash, vomiting, cardiotoxicity, neutropenia, obesity, and abdominal pain [1,10].

A patient can be defined intolerant to imatinib only when he develops serious adverse events (AEs) such as renal failure or liver injury (hepatic transaminase and bilirubin elevations), which require discontinuation or change of therapy [11].

### 2.2. Second Generation TKI

Dasatinib (Figure 2B) is the first second generation TKI approved by FDA in 2006. It is able to inhibit BCR-ABL both in the DGF in (Figure 1B) and in the DGF out conformation and also other TK such as PDGFR and Src [12]. Based on X-ray analysis, it was observed that dasatinib binds the ABL protein in a less strict conformation compared to imatinib. Despite the lower number of binding interactions observed for dasatinib vs. ABL with respect to that occurring between imatinib vs. ABL. The former is provided with inhibiting activity 325-fold higher and it is able to overcome many BCR-ABL mutations resistant to imatinib with the exception of the T315I one [13]. Furthermore, dasatinb is not P-gp substrate and, thus, can be used in patients resistant to imatinb and nilotinib.

Nilotinib (Figure 2C) is another second-generation TKI. It is a BCR-ABL, c-kit, and PDGFR inhibitor from 10-fold to 30-fold more powerful than imatinib on the BCR-ABL oncoprotein. It inhibits the proliferation of cells exhibiting the mutated TK. Nilotinib binds the protein in its inactive conformation (DFG OUT) through interactions similar to those observed for imatinib, such as four hydrogen bonds and many Van der Waals contacts. The successful introduction of the trifluoromethyl group on the phenyl ring and the substitution of the methyl-piperazine with imidazole are responsible for its greater activity. It can also overcome some Bcr-Abl mutations (L248V, G250E, Q252H) apart from T315I [14]. According to Redaelli et al. [15], by comparing the nilotinib activity vs. imatinib and dasatinib, the former showed a mild resistance to 13 out of 18 mutations of BCR-ABL with high resistance to T315I and E255V changes. Based on some clinical trials of phase III in 2011 (ENESTnd Study), [16] a major complete cytogenetic remission (CCR) was observed and a reduction in the percentage of patients proceeded to the accelerated phase of CML after an annual treatment with nilotinib in comparison with imatinib. For these reasons, nilotinib was approved as first-line therapy for newly diagnosed patients with CML in 2011.

Ponatinib (Figure 2D) is a very powerful third-generation pan-inhibitor of TKs. It was synthetized as a ligand of ATP binding domain of BCR-ABL in closed conformation. Ponatinib binds the DFG OUT conformation of the protein. The peculiarity of this molecule lies in the occurrence of a triple bond, which allows us to mitigate the steric hindrance due T315I mutation. The triple bond between the phenyl and the heterocycle ring in ponatinib can hold the isoleucine side chain. Having said that, ponatinib can be considered as a TK inhibitor unaffected from T315I mutation. Its binding is ensured by five hydrogen bonds. Moreover, there are several Van der Waals interactions supporting affinity in the case of multiple mutations [17]. Notably, ponatinib kept its activity in other CML mutations, such as M244V, G250E, and Q252H. However, its use drew attention to the appearance of an important side effect, which is the inhibition of parental cells Ba/F3 proliferation [18].

## 3. CML in Pediatric Age

**Figure 3:**
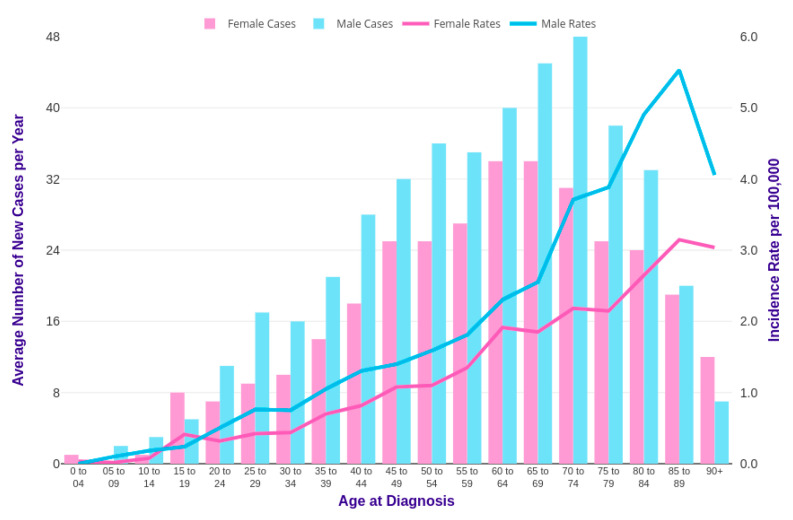
CML incidence rates in the United Kingdom (UK) [24].

Horibe et al. combined and analyzed data from more than 3856 Japanese children and 1803 young adults up to the age of 29 years with leukemia. Among pediatric leukemic patients under 15 years of age, the proportion of patients with CML was about 1.8% [19]. The relative incidence of CML starts to increase in the last teen years, between the ages of 15 and 19, up to 8.3%. Specifically, the average annual incidence of CML in children younger than 15 years is 0.6–1.0 cases per million and, for patients between 15 and 19 years of age, it is 2.1 per million [20]. Several published reports on pediatric CML have shown a male predominance of the disease with a male:female ratio of 1.3:1.7 [21,22], as reported in studies conducted on adults [23]. The incidence of CML among males and females, comparing statistics by age in the UK, is reported in Figure 3 [24]. As shown, CML mainly affects old people with a median age of CML diagnosis from 60 to 65 years, especially in western countries, [25] while it is considered a rare condition in adolescents and an ultra-rare condition in children. Only 2% of all leukemias in children younger than 15 years of age and 9% of all leukemias in adolescents between 15 and 19 years, with an annual incidence of 1 and 2.2 cases per million in these two ages groups, respectively, can be ascribed to CML [26]. As shown in Figure 3, 23% of new cases occurred in the United Kingdom (UK) in the period from 2014 to 2016, which were relative to people aged more than 75 years old. Incidence rates are significantly lower in females than in males [24] (Cancer research UK).

**Figure 4:**
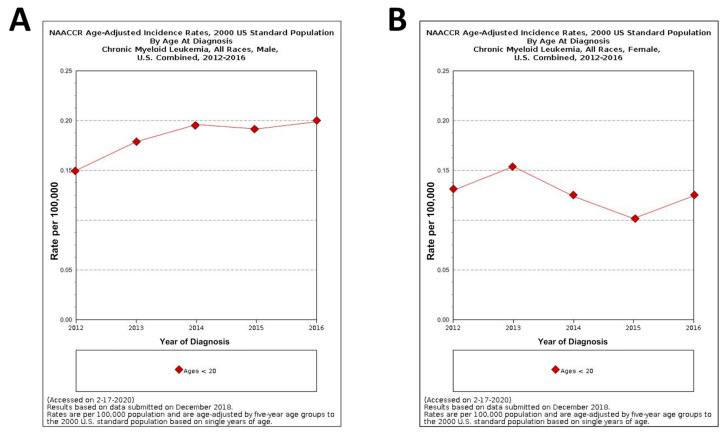
North American Association of central cancer registries (NAACCR) Fast Stats: age-adjusted incidence rates of CML in male and female patients under 20 years of ages (**A**) and (**B**), respectively [27].

The graphs in Figure 4 show the NAACCR (North American Association of central cancer registries, 2019) age-adjusted incidence rates of CML in male and female patients under 20 years of age [27]. It goes without saying that, in spite of the rareness of pediatric CML, its incidence from 2012 and 2016 is, to some extent, steadily increased in young males while a more fluctuating trend is observed in young females during the same lapse of time.

First of all, it must be considered that CML in children and adolescents is different from CML in adults. Pediatric CML shows more aggressive features, such as larger spleen size in proportion to body weight, higher white blood cell and platelet counts, and more frequent diagnosis of advanced phases [28]. There are also some genetic differences between pediatric and adult CML such as a higher number of mutations prompting cancer progression. For example, about 60% of pediatric patients have ASXL1 mutation compared to only 15% of adults [29]. These differences between pediatric and adult CML are related to host factors and CML cell biology, which leads to different clinical presentation and disease spread [28]. However, in the absence of a representative sample of cases of pediatric CML, there are currently not enough clinical studies to establish practice standards for treating this serious disorder. For this reason, many pediatric oncologists can only follow the ELN (European Leukemia Net) [28] and NCCN (National Comprehensive Cancer Network) [30] guidelines, designed for adult patients.

The scoring system commonly used to prognosticate and manage CML in adults such as SOKAL (Sokal relative risk score), Hasford (Hasford relative risk score), and EUTOS (European treatment and outcome study score), are not applicable to pediatric patients [31,32]. The International Registry for Chronic Myeloid Leukemia (I-CML-Peds study) in Children and Adolescents, after evaluating and comparing the risk group allocations and outcome between the prognostic scores in a population of 350 pediatric patients, proved that the ELTS (EUTOS Long Term Survival) score was the best metric in terms of differentiation of progression-free survival [31]. However, more data are necessary to extend and confirm ELTS applicability to children and adolescents.

### 3.1. TKI Pediatric Therapy: Issues and Concerns

Since the development of TKIs, which have replaced what is now the third line CML treatment, the allo-HSTC, life expectancy for adult patients with CML has grown significantly. However, TKI therapy for pediatric patients can be considered a thorny matter because children are actively growing during the therapy, so they can face a growth disturbance, which is an adverse event never seen in adults [33]. Moreover, they have a much longer life expectancy than adults and, consequently, should experience a longer exposure to TKI (some children must follow TKI therapy for all life). However, long-term effects of TKIs beyond 15 years of age are still missing [34]. It was, thus, wise to undertake an accurate monitoring of the length of therapy to identify the appearance of resistance or intolerance phenomena. To minimize TKI-related side effects, a discontinuation approach has been challenged in pediatric patients as done in the case of adults with a deep and sustained molecular response [28,35,36]. Unfortunately, the only discontinuation reported for children and adolescents are due to poor adherence of the therapy. In general, only a few pediatric patients discontinued TKI successfully [37,38]. As a result, there is an urgent need for perspective studies based on a much larger number of pediatric CML cases.

The goals of CML therapy are the same for adults and children, which include disease remission, reduced risk of progression, and survival. However, the treatment of pediatric CML must take into account the challenge of achieving these goals while minimizing toxicities for six or seven decades. [39] Although cure is the ideal goal for all patients regardless of age, for older adults, it may be sufficient to approach CML as a chronic disease with the aim of maintaining patients in CML-CP (Chronic Phase) for a few decades with TKIs. In the GIMEMA (Gruppo Italiano Malattie ematologiche) study, [40] the percentage of young adults who were treated with TKI and had cumulative probability of progression to AP (Accelerated Phase) and BP (Blast Phase) at 8 years was 16%, which was higher than in adults or elderly equal to 5% and 7%, respectively [28,36].

As discussed before, prolonged treatment with TKIs has potential long-term effects and, thus, different undesired effects in growing children when compared to adults. These can be cumulatively higher in the pediatric CML population in case of lifetime exposure to TKIs. Furthermore, reports from pediatric oncologists observed poor adherence more frequently in adolescents than older adults or younger children. For these reasons, extended use of TKIs is a less viable option in these patients, which makes the issue even more complicated. When selecting a TKI, it is important to consider the adherence of patients to the therapy. Twice-daily dosing of nilotinib may be more challenging for pediatric patients than once-daily dosing of imatinib or dasatinib. Formulations of TKIs with better palatability should be developed because they may improve adherence among pediatric patients. Other methods to improve adherence such as direct clinical supervision or various reminder systems may be needed in patients with suboptimal responses to TKIs. Another factor that needs to be considered is the cumulative cost of TKI therapy in children who may need decades of treatment, even though this may become a minor issue in the near future with the introduction of generic products that are much cheaper than the proprietary medicinal products.

### 3.2. HSCT vs. TKI in the Therapy of Pediatric CML

As already mentioned, HSCT (Hemapoietic Stem Cell Transplantation) is recommended only in CP-CML patients resistant or intolerant to TKIs, [41] even if it is the sole treatment option able to completely eliminate leukemic stem cells [42]. Clearly, children have longer life expectancies, and the perspective to receive TKIs lifelong could increase their risk of morbidities and, unfortunately, decrease their life quality [33]. If an appropriate donor is available, HSCT could be considered resolutive in pediatric CML by avoiding the need for prolonged TKIs and reducing the therapy cost.

Recently, Chaudhury et al. evaluated outcomes, reported in the CIBMTR (Center for International Blood and Marrow Transplant Research) database in 449 CML pediatric patients who received myeloablative HCT [43].

They analyzed several parameters influencing the outcomes, such as patient age and pre-HCT TKI therapy. They evaluated a probability of 5 years of overall survival (OS) and leukemia-free survival (LFS) post-HCT of 75% and 59%, respectively, without consequences due to a difference of age or due to pre-HCT TKI therapy. Very favorable effects were obtained if HCT was performed in more recent years and if it was a Matching Sibling Donor (MSD) HTC.

The estimated LFS in pediatric CP-CML patients treated with imatinib is about 98% at 3 years with a complete hematologic response of about 98% [44]. However, a comparison among patients treated with TKIs and others receiving HCT cannot be performed because HCT could not always be a therapy option.

An important parameter to take into account for the efficacy of CML therapy is evaluating Health-Related Quality of Life (HRQOL), recently investigated in CML patients receiving imatinib or HSTC [45,46].

In 2014, Mo et al. demonstrated that HRQOL of patients treated with Identical Sibling Donor (ISD)-HSCT is comparable to that of imatinib-treated patients [47].

Based on the evidence that young patients are able to obtain better HRQOL after HSCT, which is the only lasting cure for CML, as mentioned above. It could be considered a valuable therapy option for pediatric CP-CML.

Very recently, Athale et al. of the Children’s Oncology Group CML Working Group, in the absence of pediatric CML treatment guidelines, analyzed the issues and the concerns derived from the comparison between TKI and Allo-HSCT in pediatric age and made several recommendations about the HSCT therapy [48]. Taking into account that, in recent years, HSCT in children determined fewer complications than in adults, they recommend HSCT in pediatric age when CML progresses from CP to AP or BP, when the therapy with two different TKIs fails or when the patients show intolerance or resistance to TKIs. HSCT may also be considered an option for children and young adults in case of compliance problems, but only after a deep risk/benefit analysis.

### 3.3. TKIs Discontinuation: Treatment-Free Remission as an Additional Goal in CMLTKI

The optimal responses of CML patients to TKI treatment resulted in a large increase of the life expectancy, which is a figure now approaching that of the general population [49]. However, as mentioned before, long-term use of TKIs can be responsible for more or less severe adverse events. Among these, the most remarkable and common side effects, such as fatigue and musculoskeletal pain, may affect patient quality of life (QoL) [45]. Such a similar concern, in addition to the high up-front cost of TKI treatment, is responsible for nonadherence to therapy. For all these reasons, TKI discontinuation in order to achieve treatment-free remission (TFR) seems to be the most encouraging option for CML patients showing a sustained deep molecular response (DMR). Needless to say, such considerations need to be done prior to stopping TKI treatment. First of all, it is necessary to define the stage and the course of the disease at the time of the diagnosis. Precisely, the Sokal risk score at the time of diagnosis has been identified as an important prognostic factor for successful TFR with TKI, especially with imatinib. The Sokal risk score is an index used to predict prognosis at the time of CML diagnosis before starting treatment. It can be useful to determine risk and decide on therapy guided by the NCCN guidelines for CML. According to the TWISTER (The Australasian Leukaemia & Lymphoma Group (ALLG) CML8 study, ACTRN 12606000118505) study, a high-risk Sokal score at diagnosis was associated with molecular relapse [50]. In contrast, STIM (STOP Imatinib) study reported that patients with low-risk Sokal score had an estimated survival without relapse at 18 months of about 54%, compared with about 35% and 13% in those with an intermediate and high score, respectively [51]. Another important factor to consider is the molecular response, which is a measure of treatment success in CML. To better understand what MR (Molecular Response) is, it is necessary to remind that Bcr-Abl is the final genetic marker for Ph+.

If TKI therapy is going well, first, white blood cells return to a normal level (complete hematologic response), then Ph+ cells cannot be found in bone marrow (complete cytogenetic response) with a very small amount of Bcr-Abl (major molecular response), as long as it totally disappears from the bone marrow, which achieves the deep molecular response as a sign of disease remission. Specialists may also refer to molecular response 4.5 or MR4.5. This means a 4.5-log reduction or a decrease of 10,500 times in BCR-ABL1 transcripts, which is lower than they were before treatment started. In particular, several discontinuation trials established sustained MR4 for at least two years as the fundamental criterion for considering treatment discontinuation, [52] even though the specific eligibility criteria vary across the TRF (Treatment Free Remission) trial, which leads to choosing MR4.5 as a benchmark. Furthermore, it is necessary to clarify that the duration of DMR (Deep Molecular Response) may be more important that the duration of therapy in order to choose TKI discontinuation, as shown in the European Stop Tyrosine Kinase Inhibitor Trial (EURO-SKI), which points out that the duration of response was the most relevant factor [53]. Once the necessary criteria for TKI discontinuation have been met, why should patients choose to cease TKI therapy? Above all, they may be motivated by prospects for a better life. In particular, the mitigation or the disappearance of adverse events experienced during the treatment may improve patient quality of life (QoL). This is a fundamental concern for both children and female patients of childbearing potential, so they could particularly benefit from TFR as a treatment goal due to the adverse outcome they may experience [54,55,56]. On the other hand, discontinuing TKI can be responsible for a withdrawal syndrome, characterized by musculoskeletal pain that frequently improves spontaneously or with anti-inflammatory agents, but occasionally (albeit rarely) may require resumption of TKI therapy [57,58]. Then, there is not only an economic consideration to do in choosing the option of TKI discontinuation because it is a question of such importance as the emotional and cognitive components since patient wishes and fears must be considered and respected. All these factors help to motivate adherence to therapy discontinuation when circumstances permit it. Many trials in the past years have investigated outcomes for TFR as a treatment goal in CML. Worthy of mention is the STOP-2G-TKI study, which enrolled patients who achieved MR4.5 after receiving 2G-TKIs (dasatinib or nilotinib) for at least two years [59]. During the treatment-free phase, there was no progression to AP and all relapsing patients regained MMR (Major Molecular Response) and MR4.5 after restarting therapy. Lots of trials are currently ongoing in order to verify if it is possible to lower the standards for TFR (responses less than MR4.5 sustained for less than two years) in order to increase the pool of eligible patients. In addition, before deciding on TFR, it is necessary to accurately monitor levels of BCR-ABL1 transcripts and continue to check once treatment has stopped. The molecular monitoring could give information about a potential relapse so that patients can restart TKI immediately. The most sensitive and accurate methods for quantifying transcripts are Real-time Quantitative Polymerase Chain Reaction (RQ-PCR) or digital PCR technology. European Society for Medical Oncology (ESMO) [60] guidelines highlight the need for a rapid turnaround of PCR test results within four weeks and the capacity to provide PCR tests every four–six weeks when required, which are both feasible only in a suitably equipped laboratory [60]. Current NCCN guidelines suggest monthly monitoring for the first 12 months after discontinuation of TKI, every six weeks during months 13–24, and every 12 weeks thereafter [52]. Considering the low cost and the minimal discomfort of continued molecular monitoring, if the laboratories are capable of returning results rapidly, ideally in less than four weeks, even the patient compliance will be guaranteed. Even if lots of ongoing trials seem to prove that TKI discontinuation is a great strategy for CML management, it became evident that, while some patients show a steady increase of transcript levels after discontinuation, in many instances, low transcript levels remained. According to NCCN and ESMO guidelines, this has led to the notion that relapse, and, thus, reinitiating therapy, should be considered when MMR is lost in most instances. Understandably, further studies on TKI discontinuation are needed to help define the best predictive factors for identifying the most appropriate patients, especially for children and adolescents, because the limited available data are mainly based on case reports of noncompliant patients [61]. Therefore, current adult guidelines for stopping TKI cannot be applied to pediatric patients without proper prospective clinical trials.

### 3.4. Imatinib in Pediatric CML

Glivec (that is imatinib mesylate) is a tyrosine kinase inhibitor previously approved by the FDA for treating CML and for treating patients with Kit (CD117) positive metastatic and/or unresectable malignant gastrointestinal stromal tumors (GIST). Glivec was also approved in 2003 for use in children with Ph+ chronic phase CML, which was recurrent after stem cell transplantation or resistant to INF-α therapy. On January 2013, Glivec was also approved by FDA to treat children newly diagnosed with Philadelphia chromosome positive (Ph+) acute lymphoblastic leukemia (ALL) [62].

Since its introduction as a selective BCR-ABL1 TKI, imatinib replaced the CML first-line curative treatment, the allo-HSTC [63]. Similar to the strategy in adults, imatinib soon became the recommended initial standard of care in pediatric patients [64].

The results of clinical trials with imatinib in the adult patient population have been transferred to children, and, thus, imatinib is now the front-line treatment for childhood CML. Since the approval by the US FDA in 2003, several reports have been published on the effects and toxicity of imatinib in children. With regard to its dosage, pediatric doses of 260 mg/m^2^ and 340 mg/m^2^ have been found to be on par with 400 mg and 600 mg, respectively, in adults. Therefore, the recommended starting dose in children is 300 mg/m^2^ once daily (maximum absolute dose, 400 mg) and 400–500 mg/m^2^ for advanced-stage disease [64,65]. The lack of any specific guideline for children prompts the consideration of such adult-based criteria to measure the response and decide upon clinical status such as imatinib resistance, intolerance, noncompliance, or disease progression. In patients with an optimal response, imatinib may be continued until allo-HSCT is undertaken. In case of failure, second-generation TKIs and allo-HSCT need to be considered [66].

However, even if clinical experience with imatinib in the pediatric population is limited, a phase I trial conducted by the Children’s Oncology Groupp [65] showed that a daily oral dose of imatinib (260–570 mg/m^2^) was well tolerated in children [67]. A European group reported a multi-center phase II study of imatinib in 30 children with CML showing comparable results with those achieved in adult patients [68]. In addition to these studies, a phase III trial was conducted with 156 patients (91 male, 65 female, median age 13.2 years, range 1.2–18.0) with a newly diagnosed CML, recruited from March 2004 until December 2015 [37]. On the basis of this phase III trial, it was concluded that imatinib at the recommended dose results in both an excellent response and tolerable side effect rates in children and adolescents with newly diagnosed CML. First-line imatinib treatment allows patients to avoid major consequences of allo-HSCT, such as transplant-related mortality and chronic graft-versus-host disease. However, only careful follow-up on long-term administration of imatinib will provide information on the sustainability of responses, rates of resistance development, and treatment-related complications, especially when transition from a pediatric setting into adult hematology becomes mandatory [69,70]. Worth of mention is also a French national phase IV trial [22]. The results of this study pointed out a high rate of progression-free survival (98% at 36 months) and the achieving of CHR (Complete Hematological Respnse) for 43 patients (98%). Moreover, during the follow-up, 25 patients (75%) achieved an MMR. No treatment-related death occurred and toxicities were generally reversible with temporary treatment discontinuation or dose reduction. The proportion of patients discontinuing imatinib (30%) is very similar to that reported for adults (from 25% to 28%) [71,72]. In conclusion, this study focused on a median follow-up of 30 months that shows a satisfactory rate of response with acceptable adverse effects of Imatinib as initial therapy in children and adolescents with newly diagnosed CML in CP (Chronic Phase), which confirms its effectiveness in children and adolescents with CML in CP with response rates similar to those reported in adults. However, longer follow-up studies are necessary to have a more comprehensive view about long-term exposure [22].

As mentioned above, several studies have indicated that imatinib has a negative impact on growth and development in children with CML. In this respect, growth retardation, [33] dysregulation of bone remodeling [73], and alterations in bone metabolism [74] have been associated with imatinib treatment. A retrospective study reported significant growth deceleration after 12 months of first-line Imatinib therapy in pediatric patients [75].

Moreover, TKI response rates vary among different individuals in which pharmacokinetics is a key factor for successful CML treatments. Adherence to imatinib intake may be the most prominent factor influencing treatment outcome in teenagers, which points towards the potential benefits of regular drug monitoring [76].

### 3.5. Dasatinib in Pediatric CML

From 25% to 29% of patients that discontinued imatinib due to a poor response or toxicity [22,77] were left without approved therapies to treat children with Imatinib resistant/intolerant CML-CP.

On November 2017, the US FDA granted regular approval to the second generation TKI Dasatinib (SPRYCEL, Bristol-Myers Squibb Co.) for treating pediatric patients with Ph+ CML in the chronic phase. It is now available in tablet formulation [78].

In order to evaluate the safety and the efficacy of dasatinib in pediatric patients, many trials are still needed. Among the studies already done, a phase I trial aimed at determining suitable dosing for children with Ph+ leukemias. CA180–226/NCT00777036 is a phase II, open-label, non-randomized prospective trial of patients, 18 years of age receiving dasatinib [79]. Major cytogenetic response of >30% was reached by 3 months in the imatinib resistant/intolerant group and CCyR > 55% was reached by 6 months in the newly diagnosed CML-CP group. CCyR and major molecular response by 12 months, respectively, were 76% and 41% in the imatinib resistant/intolerant group and 92% and 52% in the newly diagnosed CML-CP group. Progression-free survival by 48 months was 78% and 93% in the imatinib resistant/intolerant and newly diagnosed CML-CP groups, respectively. No dasatinib-related pleural or pericardial effusion, pulmonary edema, or pulmonary arterial hypertension was reported. Bone growth and development events were reported in 4% of patients. Based on this trial, it was demonstrated that dasatinib is effective for treating pediatric CML-CP with a safety comparable in both children and adults [79].

### 3.6. Nilotinib in Pediatric CML

After imatinib and dasatinib, on March 2018, the FDA approved nilotinib (TASIGNA, Novartis Pharmaceuticals Corporation) for pediatric patients one year of age or older with newly diagnosed Ph+ CML-CP or Ph+ CML-CP resistant or intolerant to prior tyrosine-kinase inhibitor (TKI) [80]. Afterward, the European Medicine Agency (EMA) approved nilotinib for pediatric patients as well.

A phase II trial was conducted when imatinib was the only TKI indicated for pediatric patients with Ph+ CML-CP, with this implying alternative treatment options particularly for patients developing resistance/intolerance (R/I) to imatinib. This phase II study enrolled pediatric patients with either Ph+ CML-CP R/I to imatinib/dasatinib or newly diagnosed Ph+ CML-CP. The trial confirmed the clinical activity of nilotinib 230 mg/m^2^ twice per day in pediatric patients with newly diagnosed Ph+ CML-CP or R/I to imatinib or dasatinib. Nilotinib was associated again with a manageable safety profile. In addition, cardiovascular events were not observed in this study and no new safety signals were identified. The most frequently reported drug-related AEs included the increase in bilirubin, alanine aminotransferase (ALT), and aspartate aminotransferase (AST) as well as headache and rash [81].

Considering that, as mentioned before, the imatinib therapy could be the cause of a slow growth and a delayed puberty in pediatric CML patients, it is still necessary to verify the nilotinib safety with regard to this kind of issue.

Despite the limited size due to the rarity of CML in children, a five-year update of the randomized ENESTnd trial [16] demonstrates the efficacy of nilotinib in pediatric patients at the recommended 230 mg/m^2^ twice per day dose as well as a manageable safety profile comparable to that of Nilotinib in adults with Ph+ CML-CP [82,83]. Based on these results, nilotinib 230 mg/m^2^ twice per day has been approved in Europe [84] for treating pediatric patients with R/I or newly diagnosed CML-CP. Nilotinib can be seen as a valuable additional therapeutic option for treating pediatric CML, according to its efficacy and manageable safety profile in children.

The safety profile of nilotinib in pediatric patients was generally similar to that observed for adults. As such, no new safety signals were identified.

Therefore, a nilotinib phase II study was conducted in 2019. Nilotinib demonstrated efficacy and a manageable safety profile in pediatric patients with Ph+ CML-CP [81]. A grade 1 SAE (Severe Adverse Event) of hormone deficiency that was suspected to be related to nilotinib treatment was reported in the R/I cohort, even though slowing of growth had already been observed when the patient was receiving first-line treatment with imatinib. However, because of the limited number of patients and short follow-up period for this analysis, few conclusions can be drawn regarding the impact of nilotinib of these parameters [81].

### 3.7. Ponatinib in Pediatric CML

An important mutation in CML, the threonine-to-isoleucine mutation at position 315 (T315I), has resulted in the development of ponatinib, which is currently the only TKI effective against T315I CML. However, it has not been studied sufficiently in children [85]. As a matter of fact, ponatinib, which is the TKI most recently approved, has demonstrated efficacy in adult patients with refractory CML but comes with an increased risk of arterial hypertension as well as serious arterial occlusive and venous thromboembolic events in adults [86]. As already mentioned, mutations of the BCR-ABL1 kinase domain may cause TKI resistance and mutation screening is recommended in patients with a poor response (primary resistance) as well as those who lose the initial response (secondary resistance) [87]. A sharp and sudden increase in the BCR-ABL1 transcript ratio should always raise suspicion of poor adherence [61], as TKI resistance due to mutations in the ABL1 kinase domain are characterized by a slower expansion of the mutated clone. Each TKI has different patterns of inactivity against defined mutations. Therefore, the specific mutation needs to be considered when selecting a TKI [65]. Due to the fact that, only a few case reports are available regarding the use of this un-licensed drug in children and adolescents, it is necessary to mention the experience of the international registry of childhood chronic myeloid leukemia, according to which data from 11 children with CML registered to the I-CML-Ped-Study and from three children with Ph+ acute lymphoblastic leukemia (Ph+ ALL) treated with ponatinib were retrospectively collected [88]. According to the results of the trial and with the limitation of the retrospective nature of this study, ponatinib may be a reasonable additional treatment option for children with Ph+ leukemias who failed several lines of therapy. Another study reported the treatment of a young adolescent with chronic phase CML treated with ponatinib [85]. The patient was a 10-year-old male found to have a white blood cell count of 96,000 mL with 1% blasts and the (9;22) (q34,q11.2) translocation. In particular, the patient was started on Imatinib and, after 6 months, achieved a CMR (Complete Molecular Response). However, at 10 months, BCR-ABL was again detected by PCR and transcript levels increased over time. At 18 months, gene sequencing identified the T315I mutation and the patient had no HLA-matched donors [85]. While CML with the T315I mutation has been considered an indication for allogeneic stem cell transplant, this case demonstrates that Ponatinib may be a reasonable alternative treatment in children and adolescents.

Likewise, Imatinib and Ponatinib may also impair growth given the patient growth curve. As a matter of fact, during the therapy, the child has had a significant decline in height velocity. However, it is encouraging that it did not prevent pubertal development [85].

## 4. TKI Dosage Forms for Children: Formulation Considerations

Currently, no oral liquid formulation for TKIs is available on the market allowing exact dosing in children, according to a patient body weight or surface, age, and physio-pathological conditions. Oncology pharmacists face a constant challenge with young patients unable to swallow oral anti-cancer drugs. Thus, this makes extemporaneous oral liquid preparation a necessary requirement. Inappropriate extemporaneous preparations of TKIs may increase the risk of overdosing or underdosing. Based on a review of the literature, a few compounding recipes are available for these drugs, as shown in Table 1 [89]. Currently, imatinib (Glivec^®^), dasatinib (Sprycel^®^), nilotinib (Tasigna^®^), and ponatinib (Iclusig^®^) formulations as film-coated tablets or capsules are available on the market [90,91,92,93].

These oral solid dosage forms are limited by their rigid dose content and the ability of the children to swallow them. Tablets can be scored to allow splitting to reduce their size. However, this can result in inaccurate dosages within the fragmented tablets. Capsules can be opened and the contents taken to improve acceptability in children. However, the capsule contents may taste unpleasant and the bioavailability of the opened capsule may differ from that of the intact product [94].

The recommended daily doses of imatinib for the pediatric population with CML in chronic phase are 260 mg/ m^2^ and 340 mg/m^2^ [76]. Imatinib tablets are not manufactured in a dosage form suitable for children, and often need to be fractioned to obtain 50 mg parts with even lower doses required in young children or infants. The drug should be taken usually in the morning after breakfast or at school, at the evening before going to sleep to reduce nausea as a side effect [95]. Since imatinib acts as a local irritant, it is recommended to take the tablets with at least 120 mL of water for children <3 years old. For young patients who cannot swallow whole tablets, the tablet should be grounded and the resulting powder should be dispersed in water or apple juice or mixed with apple puree or yogurt. The acidic pH of apple juice or puree (pH 3.5) is advantageous as the solubility of imatinib strongly increases at pH values below 6.5. However, the drug is unstable in orange juice, cola, or milk under these acidic conditions [96].

Dasatinib (Sprycel^®^), which is a second generation TKI, is also formulated as coated tablets with a dosage ranging from 20 to 140 mg [97]. Until today, this drug has been used exclusively for treating adult patients, but clinical trials have shown its potential use in the treatment of CML in children, where its pharmacokinetic parameters of absorption and elimination time were comparable with those in adults with the same safety and efficacy profiles [22,98,99]. However, in these studies, dasatinib was administered to children in the form of tablets or crushed tablets dispersed in fruit juice.

There is a paucity of data in terms of stability, bioequivalence, and safety of these extemporaneously compounded oral formulations. Before an oral liquid formulation is prepared extemporaneously, it is essential to have an adequate understanding of the pharmacokinetic characteristics of the drug, its stability, compatibility with excipients, and palatability of the final preparation, ease of administration, and safety concerns [100,101,102,103].

TKI oral liquid formulations must be appropriate for the children in terms of dose, convenience, and acceptability to ensure compliance with the medication and, at the same time, safety and efficacy of the therapy. The design of a pediatric formulation needs to take into account the differences in pediatric anatomy and physiology (especially in newborns and infants) to ensure that the pharmacokinetic profile of the drug is not compromised.

Formulation can lead to differences in pharmacokinetic profiles for a drug by highlighting the risks associated with manipulating to enable administration to children. Prescribers need to be aware of the consequences of manipulating medicinal formulations, particularly for anti-cancer drugs with a narrow therapeutic index, even in extemporaneous compounding by an oncology pharmacist, where there is insufficient evidence on product quality and safety.

The choice of excipients represents another major factor involved in extemporaneous oral liquid preparation for children. The excipients used in pediatric formulations need to be appropriate for the age to minimize excipient toxicity. Usually, the major barrier in development of oral liquid formulations is taste-masking of the active ingredient. Drug taste and its palatability are the greatest barriers for completing a treatment. The excipients used in the development of a pediatric liquid formulation need to be safe and acceptable. Excipients are typically used to improve palatability, shelf-life, and/or manufacturing processes [104,105,106,107].

## 5. Conclusions

The therapeutic approach to CML has changed since the introduction of the TKI imatinib, followed by dasatinib, nilotinib, which was approved for use in children, and ponatinib, which became the first-line treatment in adults and expanded the therapeutic options, pushing allogeneic stem cell transplantation to a third-line treatment for most pediatric cases. Unfortunately, the selection of a TKI continues to rely on clinical experience in adults [108] without sufficient data on efficacy and safety specific to pediatric patients.

The availability of three TKIs is challenging to choose a first-line option, but it also gives clinicians additional treatment chances in case of a suboptimal response [108].

More experience has been gained about efficacy, toxicity profiles, and comorbidities of Imatinib compared to the other TKIs [22,37,109]. The appearance of resistance or intolerance phenomena has required the development of a second generation of TKI. Drug availability, ease of administration, and financial issues should also be considered.

Evidence-based recommendations have been established for treating CML in adults treated with TKIs. Appropriate guidelines are, however, difficult to extend to pediatric patients given the very rare occurrence in children and adolescents [105]. As a matter of fact, the CML incidence increases with age, from 0.09/100,000 at ≤ 15 years of age to 7.88/100,000 at ≥ 75 years of age. Needless to say, here are several biological and clinical differences between pediatric and adult CML. Markedly increased leukocyte count and a higher incidence of splenomegaly are characteristic features at diagnosis of pediatric patients.

TKIs are designed to inhibit BCR-ABL1 kinase, but they have unfavorable effects, so-called “off-target” complications, such as growth impairment, especially important in children, because they are constantly and actively growing. Long-term morbidity due to TKIs is unknown. Furthermore, the adverse effects on growing children have not been clearly elucidated, even though the exposure period to Imatinib is relatively short. To establish the standard therapeutic management for pediatric CML, it is important to prospectively confirm the attractive outcomes obtained in adult studies via pediatric clinical trials with a careful monitoring system for TKI-induced adverse effects, especially in growing children [110].

Limited experience with very young children, the transition of teenagers to adult medicine, and the goal of achieving treatment-free remission for this rare leukemia are more significant obstacles that require further clinical investigations [48,108].

Lastly, in order to carry out a possible and viable therapy with TKIs in the pediatric age, a key role is played by developing appropriate formulations specifically customized toward this kind of patients, since these drugs are often available in solid dosage forms, which are difficult to administer in children.

## Figures and Tables

**Table 1:** Instructions to prepare extemporaneous oral liquid suspensions of tyrosine kinase inhibitors (TKIs) [94].

TKI	Instructions
**Imatinib**	Tablets may be dispersed in water or apple juice using 50 mL for 100 mg tablet or 200 mL for 400 mg tablet. The contents must be stirred until dissolved and used immediately. For children < 3 years old, it is recommended that at least 120 mL of water or food be taken to avoid esophageal irritation.
**Dasatinib**	Tablets can be allowed to dissolve over 20 min at room temperature in 30 mL of lemonade, preservative-free apple juice, or preservative-free orange juice. After ingestion, rinse the residue off glass with 15 mL of the juice and administer.
**Nilotinib**	Capsules may be dispersed in 5 mL of applesauce and ingested immediately on an empty stomach and abstain from eating for at least 1 h.

## Abbreviations

CML	Chronic Myeloid Leukemia
PK	Protein Kinase
TK	Tyrosine Kinase
TKI	Tyrosine Kinase Inhibitor
RTK	Receptor Tyrosine Kinase
NRTK	Non-Receptor Tyrosine Kinase
VEGFR	Vascular Endothelial Growth Factor
PDGF	Platelet Derived Growth Factor
Src	Sarcoma
JAK	Janus Kinase
ABL	Abelson (gene)
BCR	Breakpoint Cluster Region (gene)
PI3K	Phosphoinositide 3-Kinase
STAT	Signal Transducer and Activator of Transcription (protein)
CP	Chronic Phase
AP	Accelerated Phase
BC	Blast Crisis
Asp	Aspartate
Phe	Phenylalanine
Gly	Glycine
ATP	Adenosine Triphosphate
DFG	Aspartate-Phenilalanine-Glycine
Thr	Threonine
HSCT	Hematopoietic Stem Cells Transplantation
HCT	Hematopoietic Cell Transplantation
INF	Interferon
CHR	Complete Hematological Response
MMR	Major Molecular Response
CCR	Complete Cytogenetic Remission
Ph	Philadelphia (chromosome)
Ph+	Philadelphia positive
P-gp	Glycoprotein P
OCT	Organic Cation Transporter
PDGFR	Platelet Derived Growth Factor Receptor
UK	United Kingdom
NAACCR	North American Association of Central Cancer Registries
ELN	European Leukemia Net
NCCN	National Comprehensive Cancer Network
CI	Confidence Interval
STIM	Stop Imatinib
LSC	Leukemic Stem Cells
ALL	Acute Lymphoblastic Leukemia
CMR	Complete Molecular Response
FDA	Food and Drug Administration
EMA	European Medicinal Agency
R/I	Resistance/Intolerance
SAE	Severe Adverse Event
HLA	Human Leukocyte Antigen
CIBMTR	Center for International Blood and Marrow Transplant Research
OS	Overall Survival
LFS	Leukemia-Free Survival
MSD	Matching Sibling Donor
HCT	Hematopoietic Cell Transplantation
HRQOL	Health-Related Quality of Life
ISD	Identical Sibling Donor
TFR	Treatment-free Remission
QoL	Quality of Life
DMR	Deep Molecular Response
EURO-SKI	European Stop Tyrosine Kinase Inhibitor
ESMO	European Society for Medical Oncology
RQ-PCR	Real-time Quantitative Polymerase Chain Reaction
